# Myeloid-derived suppressor cells endow stem-like qualities to multiple myeloma cells by inducing piRNA-823 expression and DNMT3B activation

**DOI:** 10.1186/s12943-019-1011-5

**Published:** 2019-04-13

**Authors:** Lisha Ai, Shidai Mu, Chunyan Sun, Fengjuan Fan, Han Yan, You Qin, Guohui Cui, Yadan Wang, Tao Guo, Heng Mei, Huafang Wang, Yu Hu

**Affiliations:** 1 Institute of Hematology, Union Hospital,Tongji Medical College, Huazhong University of Science and Technology, Wuhan, 430022 China; 2 Cancer Center, Union Hospital, Tongji Medical College, Huazhong University of Science and Technology, Wuhan, 430022 China

**Keywords:** Multiple myeloma, Myeloid-derived suppressor cells, Cancer stem cells, piRNA, DNA methylation

## Abstract

**Background:**

Myeloid-derived suppressor cells (MDSCs) and cancer stem cells (CSCs) are two important cellular components in the tumor microenvironment, which may modify the cancer phenotype and affect patient survival. However, the crosstalk between MDSCs and multiple myeloma stem cells (MMSCs) are relatively poorly understood.

**Methods:**

The frequencies of granulocytic-MDSCs (G-MDSCs) in MM patients were detected by flow cytometry and their association with the disease stage and patient survival were analyzed. RT-PCR, flow cytometry, western blot and sphere formation assays were performed to investigate the effects of G-MDSCs, piRNA-823 and DNA methylation on the maintenance of stemness in MM. Then a subcutaneous tumor mouse model was constructed to analyze tumor growth and angiogenesis after G-MDSCs induction and/or piRNA-823 knockdown in MM cells.

**Results:**

Our clinical dataset validated the association between high G-MDSCs levels and poor overall survival in MM patients. In addition, for the first time we showed that G-MDSCs enhanced the side population, sphere formation and expression of CSCs core genes in MM cells. Moreover, the mechanism study showed that G-MDSCs triggered piRNA-823 expression, which then promoted DNA methylation and increased the tumorigenic potential of MM cells. Furthermore, silencing of piRNA-823 in MM cells reduced the stemness of MMSCs maintained by G-MDSCs, resulting in decreased tumor burden and angiogenesis in vivo.

**Conclusion:**

Altogether, these data established a cellular, molecular, and clinical network among G-MDSCs, piRNA-823, DNA methylation and CSCs core genes, suggesting a new anti-cancer strategy targeting both G-MDSCs and CSCs in MM microenvironment.

## Background

Multiple myeloma (MM) is a B-cell malignancy characterized by the clonal expansion of plasma cells within the bone marrow (BM). Although remarkable progress has been made in the treatment of the disease, relapse with drug-resistance usually occurs and MM remains largely incurable [1]. The development of refractory clones and disease relapse are partially caused by a small population of cancer stem cells (CSCs) persisting in the BM of MM; these cells possess an unlimited capacity for self-renewal and drug resistance [2]. Although the phenotype of MM stem cells (MMSCs) hasn’t arrived consensus due to large heterogeneity, several markers such as side population (SP), sphere formation capacity and CSCs core genes have been used to identify MMSCs [3, 4]. Like somatic stem cells, CSCs reside in the tumor niche, which is comprised of numerous cell types, extracellular matrix and soluble factors, helping to maintain the stem-like properties and protect CSCs from chemotherapeutics [5–7]. In the last decades, numerous studies have focused on the capacity of non-immune cells, such as mesenchymal stem cells (MSCs), endothelial cells and perivascular cells, to maintain the stemness of CSCs [8, 9]. However, the capacity of immune cells to modulate CSC behavior and cancer progression, are poorly understood.

Myeloid-derived suppressor cells (MDSCs) are a heterogeneous population of immature myeloid cells that participate in immune suppression by inhibiting T cell activation and inducing NK cell anergy [10]. Recent studies have indicated a prognostic role for MDSCs in both solid tumors and hematological malignances, such as small cell lung cancer [11], gastrointestinal cancers [12–14], melanoma [15, 16], and NK/T lymphoma [17], among others. The critical roles of MDSCs in MM were evidenced by their substantial accumulation and activation in patients with MM, as well as their potential capacity to suppress T cells and to improve the survival of patients with MM [18, 19]. Recently, MDSCs have also been identified as pre-osteoclast cells and a potential angiogenic factor to promote MM-mediated bone destruction and angiogenesis [20]. These results prompted investigations into the important roles of MDSCs in the pathogenesis and progression of MM.

Since MDSCs and CSCs are two important cellular components in the tumor microenvironment, the interactions between them may affect the cancer phenotype and patient outcomes [10, 21, 22]. Indeed, as demonstrated in the study by Cui et al., MDSCs enhance CSCs gene expression and sphere formation in patients with ovarian carcinoma [23]. In addition, Panni et al. confirmed the effects of MDSCs on promoting CSCs formation in a mouse model of pancreatic cancer [24]. Moreover, according to Peng et al., MDSCs endow stem-like qualities to breast cancer cells through the IL-6/STAT3 and NO/NOTCH signaling pathways [25]. However, the mechanism underlying the relationship between MDSCs and MMSCs remains poorly understood.

In this study, granulocytic-MDSCs (G-MDSCs), one type of MDSCs, was found to define the tumor phenotype and impact patient outcomes by regulating MMSCs. We provided novel evidence that self-renewal capacity of MMSCs was promoted by G-MDSCs. Furthermore, we identified piRNA-823, a small non-coding RNA (ncRNA) participating in MM proliferation, as one of the most important piRNAs. Finally, we used a xenograft animal model to validate our in vitro findings and further revealed that G-MDSCs significantly promoted the stemness of MMSCs and tumor growth in vivo.

## Methods

### Patient enrollment

Seventy-two patients who were newly diagnosed with MM were prospectively enrolled at Wuhan Union hospital from February 2015 to August 2018. Ethics committee approval was obtained from the Institutional Review Board of Tongji Medical College, Huazhong University of Science and Technology (Permit Number: S307). Written informed consent was obtained from each patient or volunteer. BM aspirates and peripheral blood (PB) were collected prior to the initiation of chemotherapy. The HLA-DR^−/low^/CD33^+^/CD11b^+^/CD15^+^/CD14^−^ cell subset in PB and BM was determined using a multi-parameter flow cytometry analyzer and recorded as G-MDSCs [18, 26]. The primary MM cells was separated from BM using CD138 magnetic beads, and then prepared for side population (SP) cells analysis by flow cytometer. Other related clinical data were collected from patients’ records. The cohorts were divided into two groups according to the median frequency of G-MDSCs. Overall survival was evaluated from the date of diagnosis to the tumor-related death. Survival curves were plotted using the Kaplan-Meier method, and differences in survival were assessed using the log-rank test.

### Cell lines and mice

The MM cell lines RPMI8226 and NCI-H929 were cultured at 37 °C in a 5% CO_2_ atmosphere in RPMI1640 (Gibco, Waltham, MA, USA) with 10% fetal bovine serum (FBS, Gibco). G-MDSCs from patients or healthy volunteers were purified using CD33, HLA-DR and CD15 magnetic beads (MiltenyiBiotec, Auburn, CA, USA) according to the manufacturer’s protocol, and the purity of the sorted cells was > 95% using flow cytometry. Female BALB/c nude mice (4–6 weeks) from Beijing Hua Fukang Bioscience Company were housed in a pathogen-free environment. Animal experiments were approved by the Committee on Animal Handling of Huazhong University of Science and Technology (Permit Number: S755).

### G-MDSC suppression assay

G-MDSCs were co-cultured with T cells (4 × 10^4^/mL) at different ratios in the presence of anti-human CD3 (2.5 μg/mL) and anti-human CD28 (1.25 μg/mL) for 3 days. T-cell proliferation was detected using a Cell Counting Kit-8 (Dojindo Laboratories, Kumamoto, Japan). Specifically, 10 μL of CCK-8 reagent was added to the co-culture system, and the absorbance was recorded at 450 nm 4 h after the incubation with a 96-well multiscanner autoreader. The proportions of T cells were measured by the Annexin V-FITC/PI Apoptosis Detection kit (BD, San Diego, CA, USA) according to the manufacturer’s protocol. Then, the IFN-γ, TNF-α and IL-2 levels in the culture supernatant were detected using the Human Th1/Th2 Cytokine Kit II (BD Bioscience, San Diego, CA, USA) according to the manufacturer’s protocol. Fluorescence was measured on a FACS Canto flow cytometer (BD, San Jose, CA, USA) with FACS Diva 7.0 software.

### SP cells analysis

G-MDSCs were purified via MACS (magnetic activated cell sorting) and co-cultured with MM cell lines in the 0. 4-μm Transwell system. After co-culture for 48 h, MM cell lines were harvested and incubated with Hoechst 33342 (5 μg/mL) for 90 min at 37 °C and were then analyzed using a FACS LSR Fortessa flow cytometer (BD, San Jose, CA, USA). Verapamil was used as control. Similarly, the primary CD138^+^ MM cells separated from BM were also incubated with Hoechst 33342 and then analyzed using a flow cytometer.

### Sphere formation assay

The sphere formation assay was performed as previously described with a few modifications [4]. Briefly, after co-culture with G-MDSCs for 48 h, MM cell lines were plated with a density of 1 × 10^4^ cells/well in 6-well plates (Corning, NY, USA) and cultured with RPMI1640 medium. Spheroid formation was assessed 2 weeks after seeding with a microscope (Nikon, Tokyo, Japan).

### RNA isolation and real-time polymerase chain reaction (qRT-PCR)

RNA was isolated from tissue samples and cells using TRIzol reagent according to the manufacturer’s instructions. Complementary DNAs (cDNAs) were synthesized using the PrimeScript RT reagent Kit (Takara, Dalian, China). Then Real-Time PCR was performed using a SYBR Green RT-PCR Kit (Takara, Dalian, China). GAPDH and U6 were used as internal controls, respectively. All PCR experiments were performed in triplicate using an AB 7500 FAST Real Time PCR System (Applied Biosystems, Foster City,CA, USA).

The following primers were used: Oct-4, forward primer 5′-GGT CCG AGT GTG GTT CTG TA-3′, reverse primer 5′-GCA GCC TCA AAA TCC TCT CG-3′; Sox2, forward primer 5′-GGG GTG CAA AAG AGG AGA GTA-3′, reverse primer 5′-TGT CAT TTG CTG TGG GTG ATG-3′; Nanog, forward primer 5′-TAA TAA CCT TGG CTG CCG TCT-3′, reverse primer 5′-GCC TCC CAA TCC CAA ACA ATA-3′;DNMT1, forward primer 5′-TAT CCG AGG AGG GCT ACC-3′, reverse primer 5′-TAA GCA TGA GCA CCG TTC T-3′; DNMT3A, forward primer 5′-GGA GGA CCG AAA GGA CGG A-3′, reverse primer 5′-CCC CAT TGG GTA ATA GCT CTG AG-3′;DNMT3B, forward primer 5′-GAG ATC AGA GGC CGA AGA T-3′, reverse primer 5′- CTG TCA AGT CCT GTG TGT AG-3′ and GAPDH, forward primer 5′-GGT GAA GGT CGG AGT CAA CGG-3′, reverse primer 5′-CCT GGA AGA TGG TGA TGG GAT T-3′. The primer for U6 was 5′-GGG GTG CAA AAG AGG AGA GTA-3′, and the primer for piRNA-823 was 5′-TAA TAA CCT TGG CTG CCG TCT-3′.

### Western blot

Cells were lysed using lysis buffer and extracted for 20 min on ice. Whole-cell extractprotein (30 μg) was subjected to electrophoresis on 10% SDS-PAGE gel and transferred to nitrocellulose membranes. Then membranes were incubated with the following primary antibodies: rabbit anti-Nanog, anti-Oct-4, anti-Sox-2 (dilution 1:1000, Proteintech, Rosemont, USA), rabbit anti-DNMT1, anti-DNMT3A, anti-DNMT3B (dilution 1:1000, Cell Signaling Technology, Danvers, MA, USA), and rabbit anti-GAPDH (dilution 1:2000, Antgene, Wuhan, China).

### PiRNA-823 silencing with an antagomir treatment

RPMI8226 and NCI-H929 cells lines were transfected with antagomir-823 or antagomir-NC (GenePharma, Shanghai, China) with a concentration of 100 nM using Lipofectamine 2000 (Invitrogen, Carlsbad, CA, USA). Non-transfected MM cells and antagomir-NC-transfected MM cells were used as controls. The transfection efficiency was detected by qRT-PCR.

### Measurement of global DNA methylation levels

MM cells were collected 48 h after co-culture with G-MDSCs. Genomic DNA was isolated using a TIANamp Genomic DNA Kit (Tiangen, Beijing, China) according to the manufacturer’s protocol. Then the global DNA methylation levels in each group were detected using the Methylflash Methylated DNA Quantification Kit (Epigentek, Farmingdale, NY, USA) as described previously [27].

### In vivo tumor xenograft model

RPMI8226 cells were co-cultured with G-MDSCs for 24 h and then subcutaneously injected into nude mice (*n* = 3 for each group). The tumor volume (V) was calculated using the formula: V = 0.5 × a × b^2^ (a represents the longer tumor diameters and b represents the shorter tumor diameters). IL-6, vascular endothelial growth factor (VEGF) and IgG production levels were measured using ELISAs (R&D Systems, Minneapolis, MN, USA) according to the manufacturer’s protocol. At the end of the experiment, the xenografts in each group were fixed with formalin for immunohistochemical staining with antibodies against Ki67, Bcl-2, and cyclin D1 (Cell Signaling Technology, Danvers, MA, USA).

### Statistical analysis

All values are presented as means ± standard deviations (SD). Student’s t-test was used to compare means between two groups, while ANOVA was used to compare data among three or more groups. Spearman’s correlation coefficients were computed to assess relationships between the G-MDSC frequency and side population cells in patients with MM. Statistical significance was defined as a *P* < 0.05. All analyses were performed using GraphPad software version 7.0.

## Results

### G-MDSCs are functionally and clinically relevant in MM

**Fig. 1:**
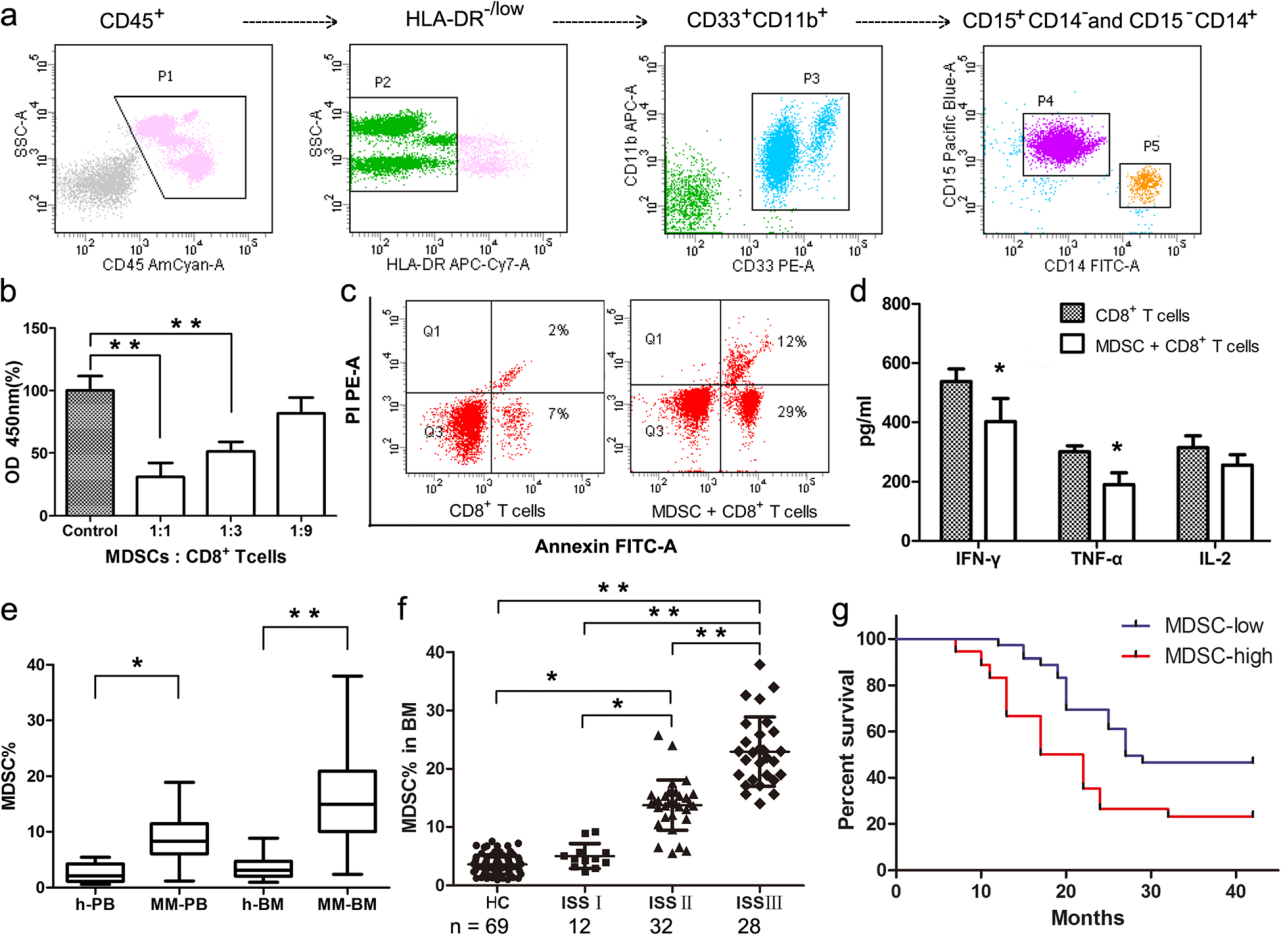
G-MDSCs are functionally and clinically relevant in MM. **a** The gating strategy for G-MDSCs in multi-parameter flow cytometry. G-MDSCs were immunophenotyped as the HLA-DR^−/low^/CD33^+^/CD11b^+^/CD15^+^/CD14^−^ population (P4, the purple cell population). **b** CD8^+^ T cells were cultured with G-MDSCs at different ratios for 72 h. Then T cell proliferation was detected using the CCK-8 assay. The data represent the means ± SD of triplicate cultures, and the results are representative of 3 different experiments. **c** Annexin V-FITC/PI staining of CD8^+^ T cells was performed 72 h after co-culture with G-MDSCs. CD8^+^ T cells were used as controls. **d** The levels of cytokines (IFN-γ, TNF-α, and IL-2) in cell culture supernatants were quantified using flow cytometry. **e** The frequencies of G-MDSCs in PB and BM from healthy donors were compared with MM patients. **f** Seventy-two MM patients were classified according to ISS. The frequencies of G-MDSCs in BM were detected, and mean values were compared between groups using the Mann–Whitney U rank sum tests. * *P* < 0.05, ** *P* < 0.01. **g** Clinical impact of G-MDSCs on the survival of MM patients. Kaplan-Meier estimates of overall survival were calculated according to the median G-MDSC density

Based on the phenotypic characteristics of G-MDSCs in hematological malignances, we identified G-MDSCs as displaying the HLA-DR^−/low^/CD33^+^/CD11b^+^/CD15^+^/CD14^−^ phenotype using multi-parameter flow cytometry analysis (Fig. 1a). G-MDSCs were isolated from the BM of MM patients for the molecular and functional studies. The results showed that G-MDSCs mediated immune suppression by inhibiting T cell proliferation, promoting T cell apoptosis and reducing cytokine secretion by effector T cells (Fig. 1b-d). Next, G-MDSCs were quantified in patients who were newly diagnosed with MM. Significantly higher frequencies of G-MDSCs were detected in both the PB (*n* = 72, mean = 8.89, *P* < 0.01) and BM (*n* = 72, mean = 15.65, *P* < 0.005) from MM patients than from healthy donors (HD) (*n* = 69, mean = 2.59 in PB and 3.47 in BM) (Fig. 1e). The frequency of G-MDSCs also significantly correlated with disease burden when stratified by the international staging system (ISS) stage, and patients with < 12% G-MDSCs exclusively harbored a low tumor burden (ISS Stage I/II) (Fig. 1f). Then the patients were divided into low and high groups based on the median G-MDSC density to examine the clinical relevance of G-MDSCs in MM patients. Overall survival (*P* = 0.004, *n* = 72, HR = 2.50, 95% CI: 1.34, 4.64) was shorter in patients with higher G-MDSC infiltration than in the lower group (Fig. 1g), indicating that an increased G-MDSC frequency is a significant indicator of poor overall survival in MM patients.

### G-MDSCs enhance stemness of MMSCs in vitro

**Fig. 2:**
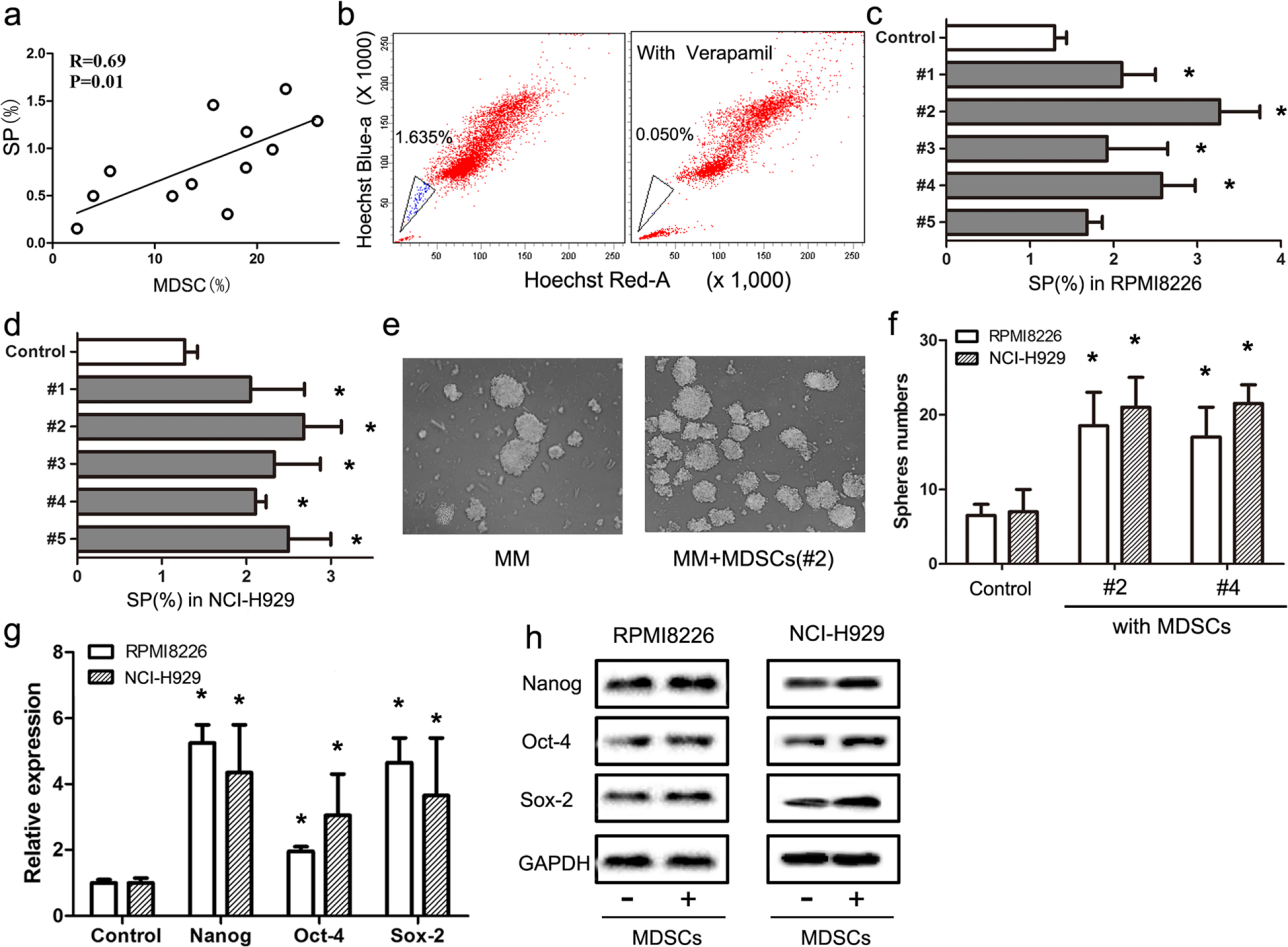
G-MDSCs enhance stemness of MMSCs in vitro*.* **a** Correlation between the proportions of G-MDSCs and SP cells in primary MM cells, as analyzed using flow cytometer (*n* = 12, R = 0.69, *P* = 0.01). **b** Representative SP cells shown using flow cytometer. Verapamil was used as negative control. **c** and **d** G-MDSCs stimulated the formation of SP cells in RPMI8226 and NCI-H929 cultures. G-MDSCs from five MM patients were co-cultured with RPMI8226 and NCI-H929 cells for 48 h. Then, the percentages of SP cells in MM cell lines were determined using flow cytometer. The results are presented as means ± SEM. * *P* < 0.05. **e** and **f** G-MDSCs co-culture increased MM sphere formation. RPMI8226 and NCI-H929 cells were cultured with G-MDSCs under sphere-forming conditions. Numbers of spheres are presented as means ± SD. * *P* < 0.05. **g** Human G-MDSCs stimulated the expression of CSCs core gene. RPMI8226 and NCI-H929 cells were co-cultured with primary G-MDSCs in Transwells for 48 h. The expression levels of CSCs core genes were quantified using qRT-PCR. The results are presented as the mean relative expression levels ± SD. **h** Levels of the Nanog, Oct-4, and Sox-2 proteins in each group were quantified using Western blotting

CSCs are regarded as the main cause of MM progression and therapeutic resistance; thus, we hypothesized that G-MDSC might affect the biological behaviors of CSCs in MM patients. SP cells enriched with CSCs were detected using flow cytometry. As shown in Fig. 2a, the frequency of SP cells among primary MM cells was positively correlated with the G-MDSCs level in the BM of MM patients (*n* = 12). Representative SP cells detected using flow cytometry are shown in Fig. 2b. Since G-MDSCs and MMSCs were correlated in MM patients, we then evaluated whether G-MDSCs promoted MM-CSC accumulation in vitro. G-MDSCs from 5 MM patients were sorted using MACS and co-cultured with MM cell lines RPMI8226 and NCI-H929. As shown in Fig. 2c, the percentage of SP cells increased during G-MDSCs co-culture. Following co-culture, RPMI8226 and NCI-H929 cells also exhibited greater sphere formation (Fig. 2d) and higher expression levels of CSCs core genes (Nanog, Oct-4, and Sox-2) (Fig. 2e and f) compared to the control. These data indicate that tumor-associated G-MDSCs promote the CSCs properties of MM in vitro.

### MDSCs enhance stemness of MMSCs via piRNA-823 up-regulation

**Fig. 3:**
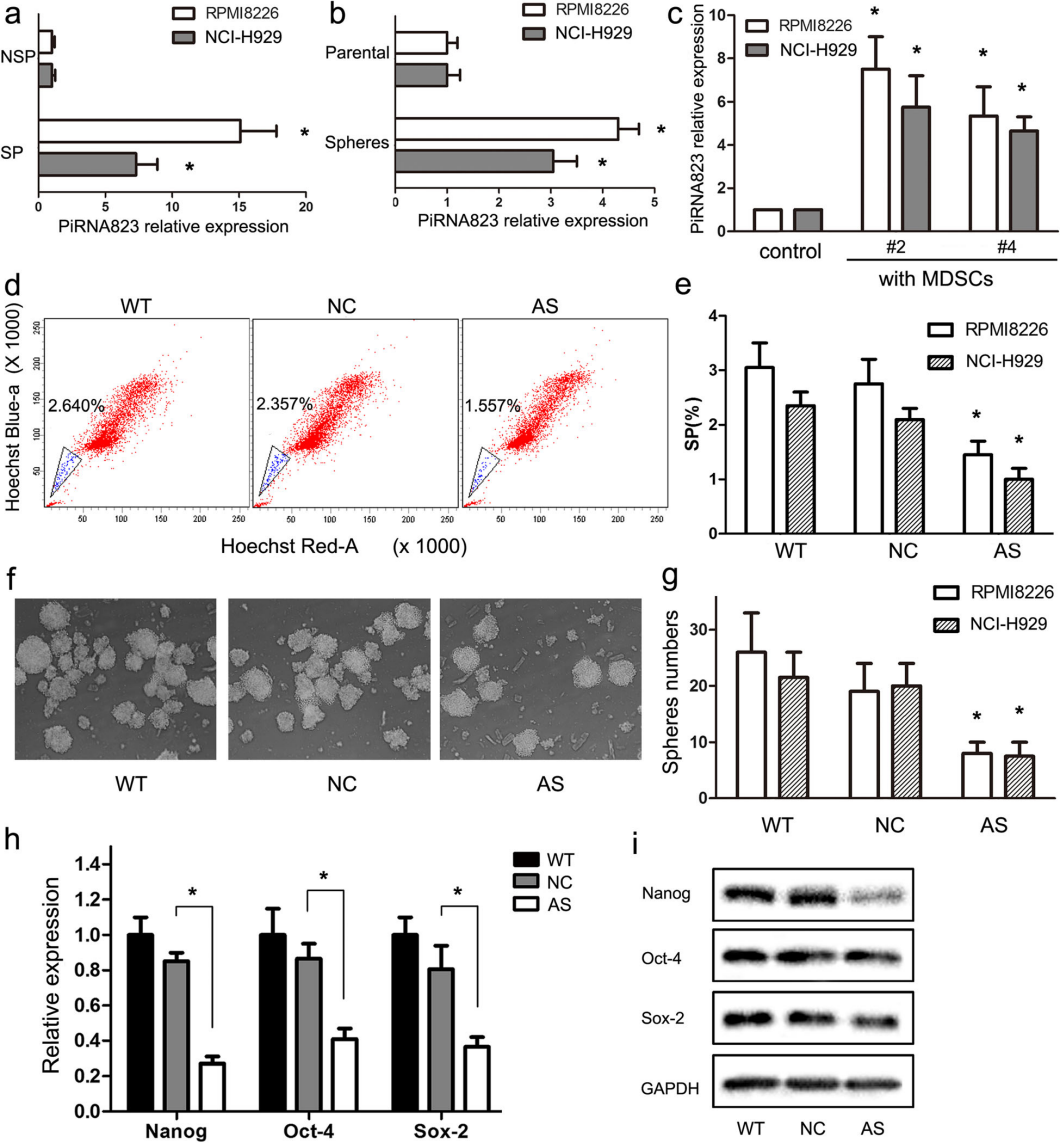
MDSCs enhance stemness of MMSCs via piRNA-823 up-regulation. **a** The expression of piRNA-823 in SP and NSP cells. SP and NSP cells were sorted from RPMI8226 and NCI-H929 cells. piRNA-823 expression was quantified using qRT-PCR. The results are presented as the mean values relative to controls ± SD; * *P* < 0.05. **b** The expression of piRNA-823 in sphere-forming cancer cells and parental RPMI8226 and NCI-H929 cells. * *P* < 0.05. **c** Effects of G-MDSCs on piRNA-823 expression in RPMI8226 cells. G-MDSCs from MM patients were co-cultured with RPMI8226 cells. piRNA-823 expression was quantified using qRT-PCR. The results from two patients (donors 2 and 4) analyzed in triplicate are shown. * *P* < 0.05. **d** Representative SP cells in each group were detected using flow cytometry. **e** G-MDSCs induced SP cell formation in MM, whereas piRNA-823 silencing markably abolished these effects. * *P* < 0.05. **f** Representative microscopy images of sphere cells in each group. **g** G-MDSCs promote sphere cells formation, whereas piRNA-823 silencing in MM cells markably reversed these effects. * *P* < 0.05. **h** The relevant expression levels of CSCs core genes were quantified in each group using qRT-PCR. The results from three experiments performed in triplicate are shown. The results are presented as the mean value relative to the controls ± SD; * *P* < 0.05. **i** Levels of Nanog, Oct-4, and Sox-2 proteins in each group were quantified using western blotting

**Fig. 4:**
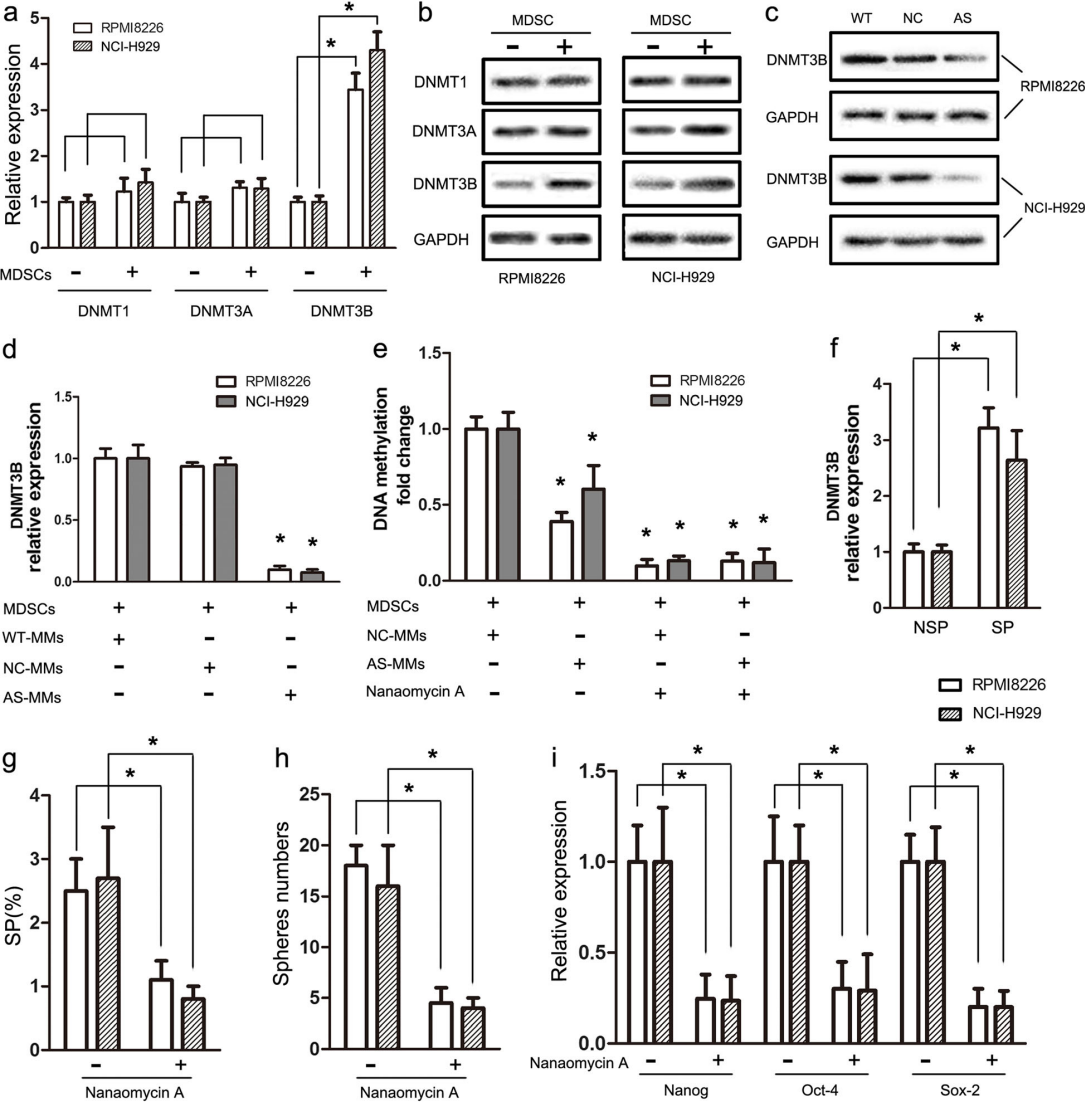
piRNA-823 controls cancer stemness by activating DNMT3B **a** RPMI8226 and NCI-H929 cells were cultured with or without MDSCs, then DNMTs mRNA levels in MM cell lines were detected by qRT-PCR. Results are presented as the mean values relative to controls ± SD; * *P* < 0.05. **b** DNMTs protein levels in RPMI8226 and NCI-H929 cells were detected by western blotting. **c** Effects of piRNA-823 silencing on DNMT3B protein levels in RPMI8226 and NCI-H929 cells. **d** Effects of piRNA-823 silencing on DNMT3B mRNA levels in MM cell lines co-cultured with G-MDSCs. * *P* < 0.05 compared to the control. **e** Effects of piRNA-823 silencing and nanaomycin A treatment on the global DNA methylation level in MM cell lines co-cultured with G-MDSCs. * *P* < 0.05 compared to the control. **f** DNMT3B mRNA levels in SP and NSP cells were quantified using qRT-PCR. SP and NSP cells were sorted from RPMI8226 and NCI-H929 cells. * *P* < 0.05. **g**, **h** Effects of nanaomycin A treatment on SP percentages and sphere formation in RPMI8226 and NCI-H929 cells. * *P* < 0.05. **i** Effects of nanaomycin A treatment on CSCs core gene transcripts expression in RPMI8226 and NCI-H929 cells. * *P* < 0.05

Next, we investigated the mechanism by which G-MDSCs enhance stemness of MM cells. We have previously demonstrated that piRNA-823 affects MM proliferation and progression [27]. However, the upstream pathway regulating piRNA-823 expression in MM are still obscure. Therefore, we firstly quantified piRNA-823 expression in MMSC-enriched myeloma cells, results in Fig. 3a and b showed higher piRNA-823 levels in SP cells and cancer sphere cells than in NSP and parental MM cells. In addition, Cui et al. reported MDSCs enhance stemness of ovarian cancer by inducing miRNA101 [23], raising the possibility that MDSCs might induce piRNA-823 expression and, in turn, promote the stemness of MM cells. In support of this hypothesis, we observed that G-MDSCs stimulated piRNA-823 expression in MM cell lines (Fig. 3c). MM cell lines were transfected with chemically modified hsa-piRNA-823 antagomir (antagomir-823) or antagomir-NC and were then co-cultured with MDSCs. As shown in Fig. 4d-g, the percentage of SP cells and number of cancer spheres formed in the antagomir-823-transfected group (AS-) were decreased compared to the antagomir-NC-transfected group (NC-) and the non-transfected group (WT-). Moreover, antagomir-823 transfection partially reversed the G-MDSCs-induced upregulation of CSCs core genes and proteins in MM cells (Fig. 3h and i), suggesting that piRNA-823 is an important factor in maintaining the stemness of MMSCs by G-MDSCs.

### piRNA-823 controls cancer stemness by DNMT3B activation

**Fig. 5:**
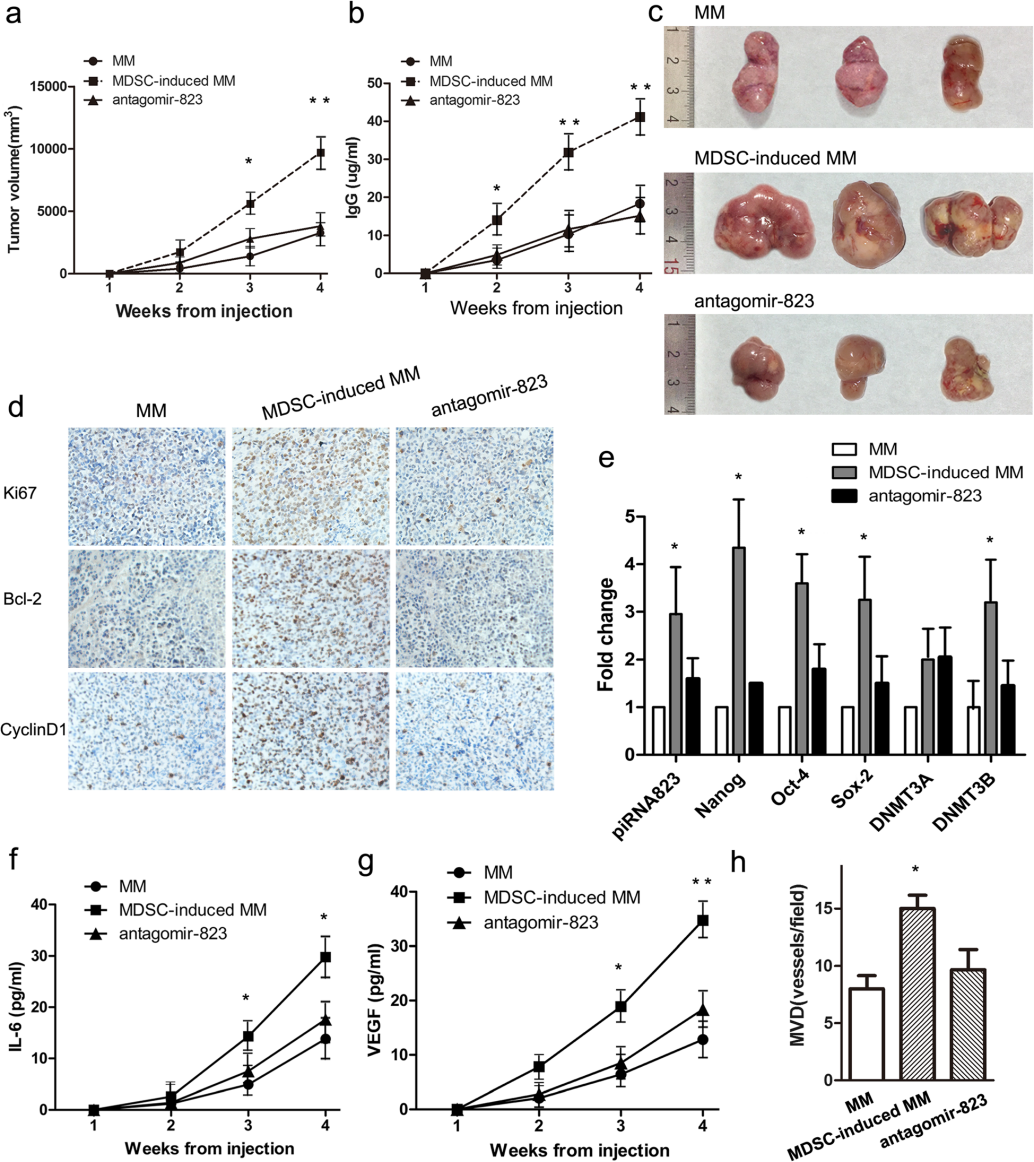
MDSCs enhance tumor growth and stemness through the piRNA-823 pathway in RPMI8226-xenografted mice. **a**-**c** MM cells, MDSCs-induced MM cells, and MDSCs-induced AS-MM cells were subcutaneously injected into nude mice (*n* = 3 for each group). Tumor volumes and IgG levels was monitored weekly. Data are presented as mean tumor volumes ± SD. * *P* < 0.05, ** *P* < 0.01. **d** Representative images of immunohistochemical staining for Ki67, Bcl-2, and CyclinD1 in tumor xenografts are shown (original magnification × 400). **e** Relative expression levels of the selected makers in tumor xenografts were detected using qRT-PCR. Values represent the means of three different experiments compared to the control ± SD. * *P* < 0.05. **f** and **g** Circulating IL-6 and VEGF levels in nude mice were detected using ELISAs. Significantly higher circulating IL-6 and VEGF levels were detected in the MDSC-induced MM group than in the other 2 groups. * *P* < 0.05, ** *P* < 0.01. **h** The quantitative analysis of microvessel density (MVD) was performed by counting the number of stained vessels in each group per high-power field (HPF) under the microscope. Data are presented as the means ± SEM of five HPFs. * *P* < 0.05

Previous studies have validated that DNA methyltransferases (DNMTs) are targets of piRNAs and play important roles in tumorigenesis [27, 28]. Therefore, we quantified DNMT1, DNMT3A and DNMT3B expression in MM cells co-cultured with G-MDSCs using qRT-PCR and Western blotting. G-MDSCs co-culture induced DNMT3B expression in RPMI8226 and NCI-H929 cells (Fig. 5a and b). Next, RPMI8226 and NCI-H929 cell lines were transfected with antagomir-823 (AS-MM) or antagomir-NC (NC-MM). Parental MM cells were defined as wild type-MM cells (WT-MM). After co-culture with G-MDSCs, DNMT3B expression levels in the WT-, NC-, and AS-MM groups were detected using Western blotting and qRT-PCR. As shown in Fig. 5c and d, antagomir-823 transfection reversed the G-MDSCs-induced up-regulation of DNMT3B in MM cell lines. Then the global DNA methylation pattern in MM cells was detected using 5-mC ELISA. Results in Fig. 5e showed that silencing of piRNA-823 in MM cell lines partially diminished the G-MDSCs-induced increment of the global DNA methylation. Nanaomycin A, a selective inhibitor of DNMT3B, significantly abolished the global DNA methylation in MM cell lines compared to controls. In addition, we measured DNMT3B expression levels in MMSC-enriched myeloma cells, and the results in Fig. 4f showed higher DNMT3B levels in SP cells than in NSP cells. Moreover, as shown in Fig. 4g-i, nanaomycin A treatment significantly reduced the SP cells percentage, spheres number and CSCs core genes expression in MM cell lines. All together, these results indicate that piRNA-823 controls cancer stemness at least partially by DNMT3B activation.

### MDSCs enhance MM growth and stemness through the piRNA-823 pathway in vivo

A human xenograft model was constructed to evaluate the effects of G-MDSCs on MM cell survival in vivo. Various numbers of CD138^+^ primary MM cells were injected into nude mice. Results showed that nearly 2,000,000 primary MM cells were needed to reach a 100% tumor incidence. However, after co-culture with G-MDSCs, only 1,000,000 G-MDSC-conditioned primary MM cells were needed to reach a 100% tumor incidence. Next, 3 types of RPMI8226 cells: WT-MM cells, MDSCs-induced WT-MM cells and MDSCs-induced AS-MM cells were injected into nude mice, which were labeled as MM, MDSC-induced MM, and the antagomir 823 group in Fig. 5 (*n* = 3 for each group). As shown in Fig. 5a-c, mice in MDSC-induced MM group had a higher tumor volume and higher IgG levels compared to the MM group. However, these effects were significantly abolished by antagomir-823 transfection. In addition, we detected proliferation-related proteins (Ki67, Bcl-2 and cyclin D1), CSCs core genes and DNMT3B levels in tumor grafts to investigate the mechanism by which antisense inhibition of piRNA-823 reduced the tumor volume in vivo. As shown in Fig. 5d and e, tumor grafts in the MDSCs-induced MM group expressed higher levels of proliferation-related proteins, CSCs core genes and DNMT3B than the control group, but these effects were significantly reversed by antagomir-823. Moreover, increased soluble IL-6, VEGF levels and microvessel density (MVD) were observed in mice in the MDSCs-induced MM group, and these effects were also significantly reversed by antagomir-823 (Fig. 5f-h). These findings suggest that silencing of piRNA-823 in RPMI8226 cells significantly decreased G-MDSCs-induced tumor proliferation and stemness in vivo.

## Discussion

The tumor microenvironment is the main battleground where tumor cells interact with immune cells. The capacity of the immune system to modulate cancer progression has been widely investigated during the last few decades [29–31]. BM microenvironment play a key role in the initiation of MM, resulting in the plasticity of MMSCs, and in turn altered tumor phenotype and function during deregulated differentiation [32]. MDSCs are one of the most important immune cell types that possess remarkable suppressive capacities by inhibiting T cell activation, inducing NK cell anergy and affecting regulatory T cell (Treg) accumulation [33–36]. In recent years, similar immunosuppressive effects of MDSCs on MM have been reported [18, 37]. However, the non-immunological effects of MDSCs on MM, particularly on MM CSCs, are relatively poorly understood.

Our in vitro observations revealed that G-MDSCs significantly increased the SP cell proportion, sphere formation, and expression of CSCs core genes in MM cells. Thus, we are the first to show that G-MDSCs directly promote and maintain the stem-like properties of CSCs in MM. Similar effects of G-MDSCs on maintaining the stem-like qualities of cancer cells have been recently reported in several types of tumors, including ovarian, breast and pancreatic cancer [23–25].These observations, combined with the results from the present study, prompted the hypothesis that cancer stemness is partially affected by immunosuppressive elements, including G-MDSCs.

piRNAs are a distinct class of small ncRNAs that maintain genomic integrity in germline stem cells. In addition to germline cells, emerging evidence has suggested that abnormal expression of piRNAs promotes tumorigenesis and even contributes to an aberrant ‘stem-like’ state [38, 39]. Here, G-MDSCs induced piRNA-823 expression in MM cells to further amplify the MMSCs pool. These effects were effectively abolished by antagomir-823, a chemically modified hsa-piRNA-823 antagomir designed to inhibit piRNA-823 expression in MM. Moreover, MDSC-induced piRNA-823 stimulation activated the DNMT3B pathway both in vitro and in vivo. Thus, we have revealed a mechanistic relationship among G-MDSCs, piRNA-823 and DNMT3B at the cellular and molecular levels in MM. The link between piRNAs and DNA methylation has been previously reported in several types of cancers [40, 41]. DNA methylation, combined with histone modification and chromatin remodeling, have also been shown to be involved in the maintenance of CSCs stemness [42]. Those observations, together with our data, suggested that the significant effects of MDSCs on the stem-like properties of MM may occur through piRNA-823 and the DNA methylation pathway. According to the results from our in vivo study, G-MDSCs increased the tumor burden and decreased survival in a tumor xenograft model. There are several possible explanations. The first is that G-MDSCs directly promoted the expansion of the MMSCs pool through the piRNA-823 pathway, as confirmed by flow cytometry and qRT-PCR in the present study. Second, IL-6 level was much higher in the MDSC-induced MM group than that in the MM group. IL-6, a critical cytokine for MM survival, was previously shown to be secreted by MDSCs in breast cancer [25]. Thus, we postulated that G-MDSCs may directly produce or induce IL-6 secretion to enhance the survival of MM cells in vivo. Third, G-MDSCs not only produced angiogenetic factors, but also were directly incorporated into the tumor endothelia, thus participating in angiogenesis [43, 44]. In the present study, elevated VEGF levels and microvessel density (MVD) were detected in the MDSC-induced MM group, indicating that MDSCs may be an important cell type mediating angiogenesis and remodeling the tumor microenvironment in MM.

Nevertheless, several limitations of this study must be carefully considered. First, only the effects of G-MDSCs on promoting MMSCs formation were examined. However, an increasing number of studies have validated important roles for M-MDSCs and tumor-associated macrophages (TAMs) in the development and progression of various cancers [45, 46]. Thus, the biological effects of M-MDSCs on MM will require further investigation in the future. Second, we only detected abnormal piRNA-823 expression in G-MDSCs-induced MM cells. Since antagomir-823 did not completely abolish the promoting effects of G-MDSCs on the tumorigenesis of MM, other piRNAs, microRNAs, lncRNAs or even circRNAs should also be considered for in-depth explorations. Third, the subcutaneous inoculation xenograft model used in this study did not completely reflect the BM milieu. Therefore, further research is required to mimic the in vivo BM microenvironment and place the interaction between G-MDSCs and MM cells in a broader context.

## Conclusions

In conclusion, G-MDSCs promote the stem-like properties of MMSCs through the piRNA823 pathway and DNA methylation. piRNA-823 silencing mostly reversed the MDSCs-induced maintenance of the stemness of MMSCs, subsequently decreasing the tumor burden in vivo. A clinical dataset also supported a correlation between the G-MDSCs frequency and overall survival of MM patients. These data suggest G-MDSCs as one of the most important cell types in the CSCs niche that promotes tumorigenesis and disease progression in MM patients. Given the importance of CSCs in tumor relapse and drug resistance, our results suggested that anti-cancer therapy should no longer regard G-MDSCs or CSCs as independent targets. Therapeutics simultaneously targeting both G-MDSCs and CSCs in the MM microenvironment may be more effective in preventing MM relapse and improving patient outcomes.

## Abbreviations

5-mC ELISA: 5-methylcytosine enzyme-linked immunosorbent assay
BM: Bone marrow
cDNAs: Complementary DNAs
CSCs: Cancer stem cells
G-MDSCs: Granulocytic-MDSCs
HD: Healthy donors
ISS: International staging system
MACS: Magnetic activated cell sorting
MDSCs: Myeloid-derived suppressor cells
MM: Multiple myeloma
MMSCs: Multiple myeloma stem cells
MSCs: Mesenchymal stem cells
MVD: Microvessel density
RT-PCR: Real-time polymerase chain reaction
SP: Side population
TAMs: Tumor-associated macrophages
Tregs: Regulatory T cells

## References

[1] Robak P, Drozdz I, Szemraj J, Robak T (2018). Drug resistance in multiple myeloma. Cancer Treat Rev.

[2] Gao M, Kong Y, Yang G, Gao L, Shi J (2016). Multiple myeloma cancer stem cells. Oncotarget.

[3] Yang Y, Shi J, Tolomelli G, Xu H, Xia J, Wang H, Zhou W, Zhou Y, Das S, Gu Z (2013). RARalpha2 expression confers myeloma stem cell features. Blood.

[4] Du J, Liu S, He J, Liu X, Qu Y, Yan W, Fan J, Li R, Xi H, Fu W (2015). MicroRNA-451 regulates stemness of side population cells via PI3K/Akt/mTOR signaling pathway in multiple myeloma. Oncotarget.

[5] Plaks V, Kong N, Werb Z (2015). The cancer stem cell niche: how essential is the niche in regulating stemness of tumor cells?. Cell Stem Cell.

[6] Zhao Yue, Dong Qiongzhu, Li Jiahui, Zhang Kaili, Qin Jie, Zhao Jiangang, Sun Qiye, Wang Zhefang, Wartmann Thomas, Jauch Karl Walter, Nelson Peter J., Qin LunXiu, Bruns Christiane (2018). Targeting cancer stem cells and their niche: perspectives for future therapeutic targets and strategies. Seminars in Cancer Biology.

[7] Kise K, Kinugasa-Katayama Y, Takakura N (2016). Tumor microenvironment for cancer stem cells. Adv Drug Deliv Rev.

[8] Papaccio F, Paino F, Regad T, Papaccio G, Desiderio V, Tirino V (2017). Concise review: Cancer cells, Cancer stem cells, and mesenchymal stem cells: influence in Cancer development. Stem Cells Transl Med.

[9] Ping YF, Zhang X, Bian XW (2016). Cancer stem cells and their vascular niche: do they benefit from each other?. Cancer Lett.

[10] Pyzer AR, Cole L, Rosenblatt J, Avigan DE (2016). Myeloid-derived suppressor cells as effectors of immune suppression in cancer. Int J Cancer.

[11] Tian T, Gu X, Zhang B, Liu Y, Yuan C, Shao L, Guo Y, Fan K (2015). Increased circulating CD14(+)HLA-DR−/low myeloid-derived suppressor cells are associated with poor prognosis in patients with small-cell lung cancer. Cancer biomarkers : section A of Disease markers.

[12] Solito S, Falisi E, Diaz-Montero CM, Doni A, Pinton L, Rosato A, Francescato S, Basso G, Zanovello P, Onicescu G (2011). A human promyelocytic-like population is responsible for the immune suppression mediated by myeloid-derived suppressor cells. Blood.

[13] Wang L, Chang EW, Wong SC, Ong SM, Chong DQ, Ling KL (2013). Increased myeloid-derived suppressor cells in gastric cancer correlate with cancer stage and plasma S100A8/A9 proinflammatory proteins. J Immunol.

[14] Gao XH, Tian L, Wu J, Ma XL, Zhang CY, Zhou Y, Sun YF, Hu B, Qiu SJ, Zhou J (2017). Circulating CD14+ HLA-DR−/low myeloid-derived suppressor cells predicted early recurrence of hepatocellular carcinoma after surgery. Hepatol Res.

[15] Weide B, Martens A, Zelba H, Stutz C, Derhovanessian E, Di Giacomo AM, Maio M, Sucker A, Schilling B, Schadendorf D (2014). Myeloid-derived suppressor cells predict survival of patients with advanced melanoma: comparison with regulatory T cells and NY-ESO-1- or melan-A-specific T cells. Clin Cancer Res.

[16] Jiang H, Gebhardt C, Umansky L, Beckhove P, Schulze TJ, Utikal J, Umansky V (2015). Elevated chronic inflammatory factors and myeloid-derived suppressor cells indicate poor prognosis in advanced melanoma patients. Int J Cancer.

[17] Zhang H, Li ZL, Ye SB, Ouyang LY, Chen YS, He J, Huang HQ, Zeng YX, Zhang XS, Li J (2015). Myeloid-derived suppressor cells inhibit T cell proliferation in human extranodal NK/T cell lymphoma: a novel prognostic indicator. Cancer immunology, immunotherapy : CII.

[18] Gorgun GT, Whitehill G, Anderson JL, Hideshima T, Maguire C, Laubach J, Raje N, Munshi NC, Richardson PG, Anderson KC (2013). Tumor-promoting immune-suppressive myeloid-derived suppressor cells in the multiple myeloma microenvironment in humans. Blood.

[19] Malek E, de Lima M, Letterio JJ, Kim BG, Finke JH, Driscoll JJ, Giralt SA (2016). Myeloid-derived suppressor cells: the green light for myeloma immune escape. Blood Rev.

[20] Zhuang J, Zhang J, Lwin ST, Edwards JR, Edwards CM, Mundy GR, Yang X (2012). Osteoclasts in multiple myeloma are derived from gr-1+CD11b+myeloid-derived suppressor cells. PLoS One.

[21] Pan Q, Li Q, Liu S, Ning N, Zhang X, Xu Y, Chang AE, Wicha MS (2015). Concise review: targeting Cancer stem cells using immunologic approaches. Stem Cells.

[22] Ugel S, De Sanctis F, Mandruzzato S, Bronte V (2015). Tumor-induced myeloid deviation: when myeloid-derived suppressor cells meet tumor-associated macrophages. J Clin Invest.

[23] Cui TX, Kryczek I, Zhao L, Zhao E, Kuick R, Roh MH, Vatan L, Szeliga W, Mao Y, Thomas DG (2013). Myeloid-derived suppressor cells enhance stemness of cancer cells by inducing microRNA101 and suppressing the corepressor CtBP2. Immunity.

[24] Panni RZ, Sanford DE, Belt BA, Mitchem JB, Worley LA, Goetz BD, Mukherjee P, Wang-Gillam A, Link DC, Denardo DG (2014). Tumor-induced STAT3 activation in monocytic myeloid-derived suppressor cells enhances stemness and mesenchymal properties in human pancreatic cancer. Cancer immunology, immunotherapy : CII.

[25] Peng D, Tanikawa T, Li W, Zhao L, Vatan L, Szeliga W, Wan S, Wei S, Wang Y, Liu Y (2016). Myeloid-derived suppressor cells endow stem-like qualities to breast Cancer cells through IL6/STAT3 and NO/NOTCH cross-talk signaling. Cancer Res.

[26] Ai L, Mu S, Wang Y, Wang H, Cai L, Li W, Hu Y (2018). Prognostic role of myeloid-derived suppressor cells in cancers: a systematic review and meta-analysis. BMC Cancer.

[27] Yan H, Wu QL, Sun CY, Ai LS, Deng J, Zhang L, Chen L, Chu ZB, Tang B, Wang K (2015). piRNA-823 contributes to tumorigenesis by regulating de novo DNA methylation and angiogenesis in multiple myeloma. Leukemia.

[28] Amodio N, Leotta M, Bellizzi D, Di Martino MT, D'Aquila P, Lionetti M, Fabiani F, Leone E, Gulla AM, Passarino G (2012). DNA-demethylating and anti-tumor activity of synthetic miR-29b mimics in multiple myeloma. Oncotarget.

[29] Dunn GP, Bruce AT, Ikeda H, Old LJ, Schreiber RD (2002). Cancer immunoediting: from immunosurveillance to tumor escape. Nat Immunol.

[30] Matsushita H, Vesely MD, Koboldt DC, Rickert CG, Uppaluri R, Magrini VJ, Arthur CD, White JM, Chen YS, Shea LK (2012). Cancer exome analysis reveals a T-cell-dependent mechanism of cancer immunoediting. Nature.

[31] Shimizu Kanako, Iyoda Tomonori, Okada Masahiro, Yamasaki Satoru, Fujii Shin-ichiro (2018). Immune suppression and reversal of the suppressive tumor microenvironment. International Immunology.

[32] Matsui W, Wang Q, Barber JP, Brennan S, Smith BD, Borrello I, McNiece I, Lin L, Ambinder RF, Peacock C (2008). Clonogenic multiple myeloma progenitors, stem cell properties, and drug resistance. Cancer Res.

[33] Gabrilovich DI, Nagaraj S (2009). Myeloid-derived suppressor cells as regulators of the immune system. Nat Rev Immunol.

[34] Li H, Han Y, Guo Q, Zhang M, Cao X (2009). Cancer-expanded myeloid-derived suppressor cells induce anergy of NK cells through membrane-bound TGF-beta 1. J Immunol.

[35] Zoso A, Mazza EM, Bicciato S, Mandruzzato S, Bronte V, Serafini P, Inverardi L (2014). Human fibrocytic myeloid-derived suppressor cells express IDO and promote tolerance via Treg-cell expansion. Eur J Immunol.

[36] Lindau D, Gielen P, Kroesen M, Wesseling P, Adema GJ (2013). The immunosuppressive tumour network: myeloid-derived suppressor cells, regulatory T cells and natural killer T cells. Immunology.

[37] Ramachandran IR, Condamine T, Lin C, Herlihy SE, Garfall A, Vogl DT, Gabrilovich DI, Nefedova Y (2016). Bone marrow PMN-MDSCs and neutrophils are functionally similar in protection of multiple myeloma from chemotherapy. Cancer Lett.

[38] Siddiqi S, Matushansky I (2012). Piwis and piwi-interacting RNAs in the epigenetics of cancer. J Cell Biochem.

[39] Mei Y, Clark D, Mao L (2013). Novel dimensions of piRNAs in cancer. Cancer Lett.

[40] Zhang W, Liu H, Yin J, Wu W, Zhu D, Amos CI, Fang S, Lee JE, Li Y, Han J (2016). Genetic variants in the PIWI-piRNA pathway gene DCP1A predict melanoma disease-specific survival. Int J Cancer.

[41] Taki M, Abiko K, Baba T, Hamanishi J, Yamaguchi K, Murakami R, Yamanoi K, Horikawa N, Hosoe Y, Nakamura E (2018). Snail promotes ovarian cancer progression by recruiting myeloid-derived suppressor cells via CXCR2 ligand upregulation. Nat Commun.

[42] Ng KW, Anderson C, Marshall EA, Minatel BC, Enfield KS, Saprunoff HL, Lam WL, Martinez VD (2016). Piwi-interacting RNAs in cancer: emerging functions and clinical utility. Mol Cancer.

[43] Yang L, DeBusk LM, Fukuda K, Fingleton B, Green-Jarvis B, Shyr Y, Matrisian LM, Carbone DP, Lin PC (2004). Expansion of myeloid immune suppressor gr+CD11b+ cells in tumor-bearing host directly promotes tumor angiogenesis. Cancer Cell.

[44] Sevko A, Umansky V (2013). Myeloid-derived suppressor cells interact with tumors in terms of myelopoiesis, tumorigenesis and immunosuppression: thick as thieves. J Cancer.

[45] Sica A, Massarotti M (2017). Myeloid suppressor cells in cancer and autoimmunity. J Autoimmun.

[46] Sica A, Porta C, Amadori A, Pasto A (2017). Tumor-associated myeloid cells as guiding forces of cancer cell stemness. Cancer immunology, immunotherapy : CII.

